# Monomorphic Epitheliotropic Intestinal T-Cell Lymphoma With Extraintestinal Areas of Peripheral T-Cell Lymphoma Involvement

**DOI:** 10.7759/cureus.10021

**Published:** 2020-08-25

**Authors:** Anoshia Afzal, Azadeh Esmaeili, Sami Ibrahimi, Umar Farooque, Bradley Gehrs

**Affiliations:** 1 Pathology, University of Oklahoma Health Sciences Center, Oklahoma City, USA; 2 Hematology/Oncology, University of Oklahoma Health Sciences Center, Oklahoma City, USA; 3 Neurology, Dow University of Health Sciences, Karachi, PAK

**Keywords:** monomorphic epitheliotropic intestinal t-cell lymphoma, peripheral t-cell lymphoma, humans, extraintestinal

## Abstract

Monomorphic epitheliotropic intestinal T-cell lymphoma (MEITL) is a primary intestinal T-cell lymphoma, previously known as enteropathy-associated T-cell lymphoma (EATL) type II. Its clinical, morphologic, and immunophenotypic features distinguishing it from the more common EATL (previously EATL type I) made it a separate entity. Unlike EATL, MEITL typically is noted in Asian, Hispanic, and indigenous populations; it is rarer in native European and Caucasian populations. Due to its poor prognosis, it needs to be distinguished from inflammatory diseases and less aggressive T-cell lymphomas. We present an unusual case of MEITL in a Caucasian patient who developed nonspecific GI symptoms and was diagnosed with MEITL of the jejunum, mesenteric lymph nodes, and multiple extraintestinal sites based on histology, immunophenotype, molecular testing, and imaging. Despite aggressive treatment, he expired about seven months after the definitive diagnosis.

## Introduction

Monomorphic epitheliotropic intestinal T-cell lymphoma (MEITL), formerly known as enteropathy-associated T-cell lymphoma (EATL) type II, is a rare peripheral extranodal T-cell lymphoma (PTCL). It arises from intestinal intraepithelial T lymphocytes and tends to behave aggressively [1]. It differs from EATL in that it predominantly affects Asian populations and is not associated with celiac disease and/or other malabsorption syndromes and inflammatory colitis. Most often MIETL involves the small bowel, particularly the jejunum and ileum, but it can also involve the stomach, colon, and other extraintestinal sites [2]. Microscopically, the tumor typically consists of small- to medium-sized monomorphic lymphocytes with hyperchromatic nuclei with inconspicuous nucleoli and a moderate amount of clear to pale eosinophilic cytoplasm. The mitotic activity is mostly brisk, and unlike EATL, there usually is no significant inflammatory background or necrosis [3]. There is a prominent epitheliotropism associated with MEITL, and it typically shows villous distortion without the villous atrophy and crypt hyperplasia often noted in the adjacent mucosa with EATL. Immunophenotyping shows that the tumor cells typically are cluster of differentiation (CD)3+, CD5-, CD4-, CD8+, CD56+, CD30-, gamma-delta T-cell receptor (GD TCR)+, alpha-beta TCR (AB TCR)-, T-cell intracellular antigen (TIA)+, megakaryocyte-associated tyrosine kinase (MATK)+, and Epstein-Barr virus (EBV) encoded small nuclear RNAs (EBER)-. About 80% of the cases show TCR-γ and TCR-δ gene rearrangements [4]. Common genetic changes include extra copies of MYC at 8q24 and gains of 9q34.3.

In this case report, we have described a Caucasian patient who presented with chronic gastrointestinal (GI) symptoms and was found to have MEITL upon resection of small intestine with multiple other areas of involvement noted by imaging. We also describe some of the features that help distinguish MEITL from other intestinal T-cell lymphomas along with a brief literature review of MEITL.

## Case presentation

The patient was a 39-year-old obese male with a history of hypertension. He presented for evaluation of left-sided abdominal pain and intermittent diarrhea. His laboratory data showed normal amylase and lipase levels and elevated lactate dehydrogenase (LDH) and alkaline phosphatase levels. Tests for Clostridium difficile, Giardia, Salmonella, Shigella, and Campylobacter were negative. Abdominal CT showed dilated, irregular segments of thickened jejunum, mesenteric lymphadenopathy, and extraluminal gas; it was interpreted as a perforated small bowel with a concern for lymphoma or another neoplastic process. He subsequently underwent small bowel resection. Histopathologic evaluation (see below) revealed a CD30-negative T-cell lymphoma, consistent with MEITL. Positron emission tomography (PET) suggested additional involvement in the bilateral adrenal glands. The staging bone marrow evaluation, including flow cytometric analysis and cytogenetic analysis, was unremarkable.

He was treated with appropriate chemotherapy. Follow-up abdominal CT evaluation after three months showed a regression of adrenal nodules and a mesenteric mass, but his PET scan revealed a new 3.9 cm nodule in the left lower lobe of his lung and persistent hypermetabolic central mesenteric nodes. An additional two months later, CT evaluation of the chest and abdomen showed disease progression with increases in the size of the lung nodule and adrenal masses. He ultimately presented to the emergency room with severe left-sided abdominal pain, diarrhea, nausea, worsening confusion, fatigue, and occasional dizziness; he was found to have hyponatremia, hypokalemia, and hypocalcemia. His clinical status continued to deteriorate, and he died seven months after diagnosis secondary to sepsis and disease progression.

Microscopic description

**Figure 1:**
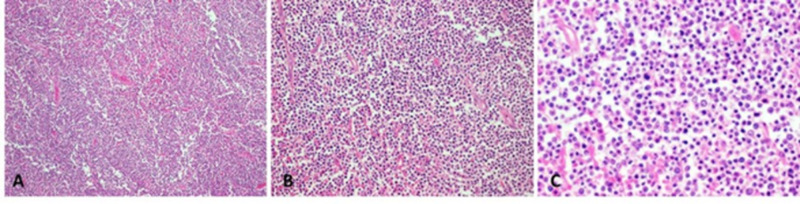
MEITL H&E (A, B, C: ×10, ×20, and ×40, respectively): There is a predominantly diffuse, infiltrate of atypical medium-sized cells with scattered mitosis and apoptosis. MEITL, monomorphic epitheliotropic intestinal T-cell lymphoma; H&E, hematoxylin and eosin

Sections of jejunum reveal a predominantly diffuse, but focally nodular, infiltrate of atypical medium-sized cells with a moderate amount of cytoplasm (Figure 1), which focally extends from the lamina propria to the serosa. Scattered apoptotic bodies are noted. There is focal ulceration. Occasional benign-appearing follicles are seen.

Immunohistochemistry and cytogenetics

**Figure 2:**
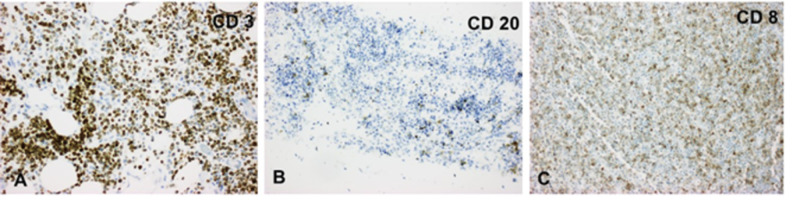
The tumor cell population was positive for CD3 (A) and CD8 (C), and it was negative for CD20 (B). CD, cluster of differentiation

**Figure 3:**
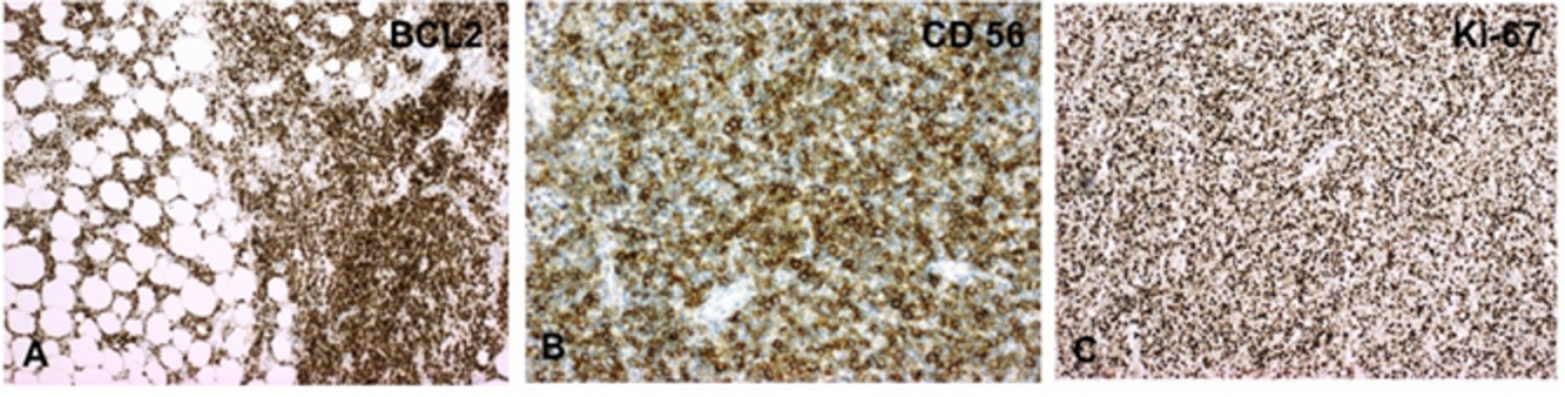
The tumor cell population demonstrated strong and diffuse positivity for BCL-2 (A) and CD56 (B), and Ki-67 (C) was positive in approximately 70% of tumor cells. BCL, B-cell lymphoma; CD, cluster of differentiation

The tumor cells were positive for CD3 (Figure 2A), negative for CD20 (Figure 2B), and positive for CD8 (Figure 2C), B-cell lymphoma 2 (bcl-2) (Figure 3A), and CD56 (Figure 3B) along with partial CD30 positivity. The tumor cells were also negative for CD23, paired-box containing 5 (pax-5), CD10, and bcl-6. Ki-67 was positive in approximately 70% of tumor cells (Figure 3C). CD5 and CD4 were positive in scattered cells within the tumor, but most of the tumor cells were negative. Most of the tumor cells also were negative for both GD TCR and AB TCR. EBER was negative. Fluorescence in situ hybridization (FISH) revealed three copies of the MYC gene in 60% of the cells.

## Discussion

This case represented a primary T-cell lymphoma with the immunophenotype features consistent with monomorphic epitheliotropic T-cell lymphoma. The World Health Organization (WHO) classification scheme of 2016 recognizes four subtypes of intestinal T-cell lymphoma: enteropathy-associated T-cell lymphoma, monomorphic epitheliotropic intestinal T-cell lymphoma, intestinal T-cell lymphoma, not otherwise specified, and indolent T-cell lymphoproliferative disorder of the GI tract (provisional). MEITL generally is positive for CD3, CD8, and CD56 and negative for CD5; the majority of cases are positive for the GD TCR, but some cases are negative for both the GD TCR and the AB TCR; extra signals of MYC (8q24) are commonly seen [1].

Inflammatory bowel disease, indolent T-cell lymphoproliferative disease, EATL, and intestinal natural killer (NK)/T-cell lymphoma can be the differentials of MEITL [5]. Although there is no association between MEITL and celiac disease, recent reports have suggested some cases of MEITL to be preceded by a variant of celiac disease [6].

MEITL is an aggressive T-cell lymphoma with a poor prognosis [7]. Unlike EATL, MEITL is less common in Northern Europeans but is more prevalent in Asian and Hispanic individuals [8]. MEITL can have endoscopic features similar to various types of colitis [5]. Histologically, it is important to distinguish it from cryptitis and microscopic colitis due to the presence of intraepithelial lymphocytes (IELs) in all of them and they can have similar endoscopic appearance [9,10]. The minimum number of IELs to be considered abnormal is 20 per 100 epithelial cells [11]. Usually, the number of IELs is far greater in lymphoma than in inflammatory conditions and there is marked cytologic atypia present [3,4]. The most important features that can help us differentiating among MEITL and other types of T-cell lymphoma are the monomorphic cell shapes, epitheliotropic patterns, and immunopositivity for CD8 and CD56.

## Conclusions

MEITL is an aggressive malignancy with a poor prognosis. Therefore, any patient presenting with vague GI symptoms, which are not explained by other pathologic processes, should be checked for MEITL as early diagnosis is essential for timely management.
